# Intelligent recognition of tobacco leaves states during curing with deep neural network

**DOI:** 10.3389/fpls.2025.1604382

**Published:** 2025-07-02

**Authors:** Qiang Xu, Yanling Zhang, Aiguo Wang, Guangqing Chen, Xianjie Cai, Shuoye Zhou, Junying Li, Baofeng Jin, Ding Yan, Jiajie Huang, Zuxiao Chen, Heng Zhang, Jianwei Wang, Weimin Guo, Jianjun Liu

**Affiliations:** ^1^ Tobacco Agricultral Labaratory, Zhengzhou Tobacco Research Institute of China National Tobacco Corporation (CNTC), Zhengzhou, China; ^2^ Tobacco Leaf Administration Office, Henan Provincial Tobacco Company of CNTC, Zhengzhou, China; ^3^ Tobacco Raw Materials Procurement Center, Shanghai Tobacco Group Co. Ltd., Shanghai, China; ^4^ Pingdingshan Branch, Henan Provincial Tobacco Company, Pingdingshan, China; ^5^ Technical Center, China Tobacco Guangdong Industrial Co. Ltd, Guangzhou, China; ^6^ Technical Center, Jilin Tobacco Industry Co. Ltd., Changchun, China

**Keywords:** tobacco leaves, large-scale dataset, bulk curing barn, image recognition, deep learning

## Abstract

**Introduction:**

The state monitoring of tobacco leaves during the curing process is crucial for process control and automation of tobacco agricultural production. While most of the existing research on tobacco leaves state recognition focused on the temporal state of the leaves, the morphological state was often neglected. Moreover, the previous research typically used a limited number of non-industrial images for training, creating a significant disparity with the images encountered in actual applications.

**Methods:**

To investigate the potential of deep learning algorithms in identifying the morphological states of tobacco leaves in real industrial scenarios, a comprehensive and large-scale dataset was developed in this study. This dataset focused on the states of tobacco leaves in actual bulk curing barn in multiple production areas in China, specifically recognizing the degrees of yellowing, browning, and drying. Then, an efficient deep learning method was proposed based on this dataset to enhance the predictive performance.

**Results:**

The prediction accuracy achieved for the yellowing degree, browning degree, and drying degree were 83.0%, 90.5%, and 75.6% respectively. The overall average accuracy, satisfied the requirements of practical application scenarios with a value of 83%.

**Discussion:**

Our proposed framework effectively enables morphological state recognition in industrial curing, supporting parameter optimization and enhanced tobacco quality.

## 1 Introduction

Curing is an important process of tobacco production, converting fresh leaves into commercially cigarette raw materials. Curing quality of tobacco leaves directly determines farmers’ income and the cigarette quality. Matching the optimal curing technology in real time according to the state of tobacco leaves is the key to determine the curing quality of tobacco leaves (Siddiqui, 2001; Zhao et al., 2024). At present, the identification of tobacco leaf states during the curing process mainly relies on people’s subjective experience. Inaccurate cognition has caused problems such as uneven curing quality of tobacco leaves, large curing losses, and weak industrial usability (Ma et al., 2021).

The rapid development of technologies such as the Internet of Things and artificial intelligence has proposed new methods for solving such problems. Researchers conducted research on the state of tobacco leaves during the curing process by using the collected temperature, humidity and tobacco leaf images. The researches on tobacco leaf state identification mainly can be divided into two categories: temporal state and morphological state. The temporal state of tobacco leaves refers to the division of the tobacco leaf curing process into different stages based on the curing time of the tobacco leaves, such as yellowing stage, color fixing stage, and stem drying stage (Li et al., 2022; Lu et al., 2023). Although this method has achieved high accuracy (More than 90%), it is difficult to adjust the temperature and humidity of the curing room in real time based on the recognition results. Therefore, some researchers focused on the morphological state recognition (Wang et al., 2017; Wang and Qin, 2022; Zhao et al., 2024). The morphological state of tobacco leaves refers to the specific state of yellowing degree, drying degree and browning degree of tobacco leaves identified based on tobacco leaf images, thereby replacing the human eye observation and subjective analysis during the curing process, and providing more accurate, faster and scientific results for identifying the state of tobacco leaves. Meanwhile, it can also provide an important reference for the real-time adjustment of the curing technology (Condorí et al., 2020; Pei et al., 2024). To further improve the recognition accuracy, the texture information of tobacco leaf images has also begun to be gradually utilized except the widely used color information (Wang et al., 2017).

However, there are still some problems limited the accuracy and application of these recognition models. Previous research on tobacco leaf state recognition often relied on small-scale (i.e., hundreds to just over a thousand samples) (Zhao et al., 2024) or non-industrial datasets, which were collected using small ovens, experimental chambers, etc (Wu and Yang, 2021; Condorí et al., 2020). For example, some studies have acquired images through smartphone photography (Howard et al., 2017; Zhang et al., 2023), but the quality and characteristics of these images differ significantly from those captured in actual curing barns, limiting their applicability to real-world bulk curing scenarios. Some researchers tried to collect tobacco images in the actual curing barns, but the complex environment during curing process resulted in image distortion, out of focus, obvious color difference and only partial tobacco image acquisition, which are still the core problems limiting the acquisition of tobacco condition information (Condorí et al., 2020; Pei et al., 2024; Wu et al., 2014; Wu and Yang, 2019; Zhang et al., 2013) and further effected the wide application of the recognition models.

To overcome these limitations, the objective of this study is to (i) construct a comprehensive and large-scale image dataset captured directly from actual bulk curing barns; (ii),propose a deep learning approach to recognize the morphological states of tobacco leaves throughout the curing process based on this dataset; (iii) establish a benchmark framework using state-of-the-art models, including the Swin Transformer V2, to enhance predictive performance and support intelligent decision-making during tobacco curing.

## 2 Materials and methods

### 2.1 Large-scale curing tobacco leaves dataset

**Figure 1:**
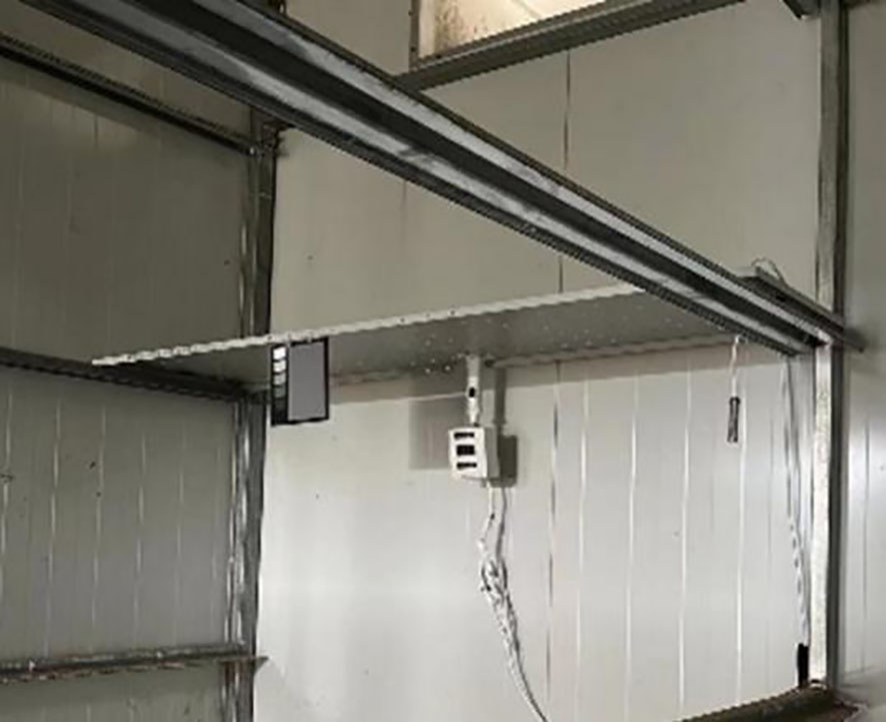
Image acquisition device and installation photos of tobacco leaves during curing process.

The large-scale curing tobacco leaves dataset involved gathering a substantial amount of real-world data from bulk curing barn and having them meticulously labeled by experts in tobacco curing. To facilitate the recognition of tobacco leaves states during the curing process, 17,420 images of tobacco leaves from 10 main production areas in China were collected, including Henan, Fujian, Yunnan, Guizhou, etc. All tobacco leaf images in the dataset were collected by a newly developed autonomous imaging device (**Figure 1**). The image device was installed in the middle shed on one side of the grill near the heating chamber in the curing barns (Xu et al., 2024). The sampling interval was set to 10 minutes, and the tobacco images of the curing process were obtained, which marked the time, location, temperature, and humidity in the curing barns and the status of the tobacco leaves.

**Figure 2:**
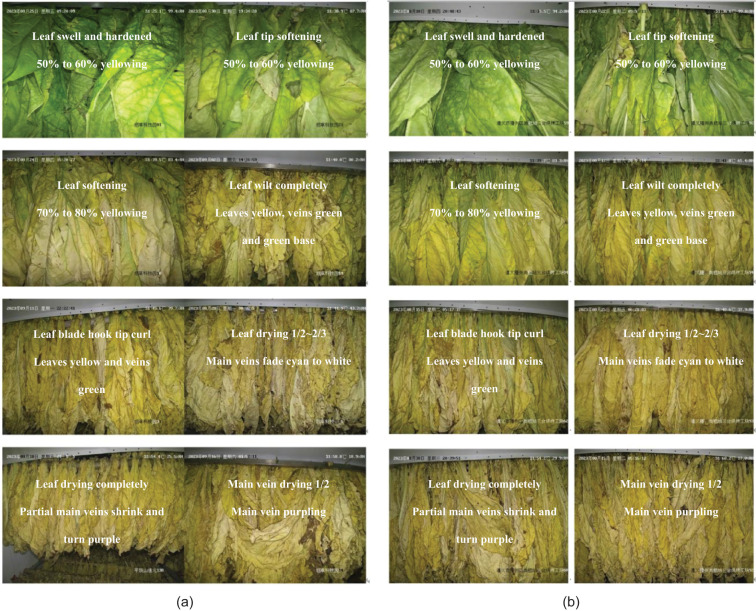
Reference image data of **(A)** Pingdingshan and **(B)** Zunyi of different yellowing and drying degrees of tobacco leaves during curing.

Experts in tobacco curing in China conducted evaluations focusing on three distinct states of the tobacco leaf: the degree of yellowing, the degree of browning, and the degree of drying. Different degrees were categorized based on the extent of morphological differences observed in the various states of the tobacco leaves (**Table 1**). In **Figure 2**, some reference images along were provided with their corresponding yellowing degree, browning degree, and drying degree labels for further clarity. This visual representation aids in understanding the various states and degrees of tobacco leaves during the curing process.

**Table 1:** The detailed definition of the states of tobacco leaf.

States	Degrees
Yellowing degree	0. 50%∼60% yellowing
	1. 70%∼80% yellowing
	2. Leaves yellow, veins green and green base
	3. Leaves yellow and veins green
	4. Main veins fade cyan to white
	5. Partial main veins shrink and turn purple
	6. Main vein purpling
Drying degree	0. Leaf swell and harden
	1. Leaf tip softening
	2. Leaf softening
	3. Leaf wilt completely
	4. Leaf blade hook tip curl
	5. Leaf drying 1/2∼2/3
	6. Leaf drying completely (Large roll)
	7. Main vein drying 1/2
	8. Main vein drying completely
Browning degree	0. 0%
	1.<10%
	2. 10%∼20%
	3. 20%∼30%
	4. 30%∼50%
	5. *>*50%

### 2.2 Recognition algorithm

#### 2.2.1 Method overview

**Figure 3:**
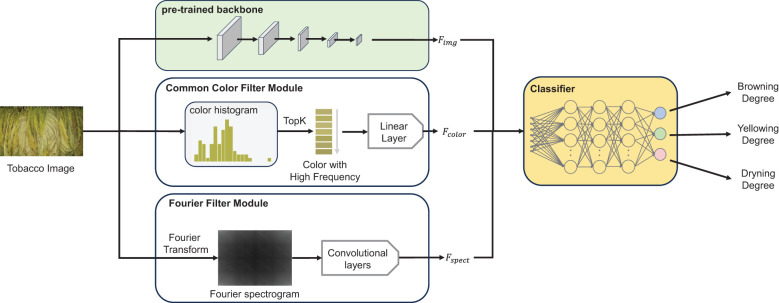
The overview of the recognition algorithm, including three key components: **(A)** a pre-trained backbone (Swin-Transformer v2), **(B)** a Common Color Filter Module and **(C)** a Fourier Filter Module. Each module was designed to extract distinct features, which were then integrated and fed into the subsequent classification network to predict the states of tobacco leaves.

A recognition algorithm was implemented to accurately and efficiently identify tobacco states during the curing process based on deep neural networks. As depicted in **Figure 3**, three components were comprised in the recognition algorithm: (i) a pre-trained backbone pre-trained on universal image recognition datasets (e.g., ImageNet), (ii) a Fourier filter module, and (iii) a common color filter module. The pre-trained backbone extracted highly discriminative image features *F_img_* by leveraging previously learned information and further fine-tunes the parameters on the proposed datasets. The Fourier filter module was designed to extract the wrinkle information of tobacco leaves *F_spect_* by utilizing the Fourier spectrum map of the image and a convolution-based network. The common color filter module calculated the quantized color histogram of the image and filtered it by frequency, thereby screening out high-frequency colors and the order of the colors appearing in the image. It further employed a fully connected layer to extract high-frequency color features *F_color_*. Finally, all features were concatenated and fed into the fully connected classifier to predict three states of tobacco.

In summary, a three-branch network was presented in this study incorporating two proposed modules: the Fourier filter module and the common color filter module. Through joint training, the network employs a multi-label recognition head to simultaneously classify three tobacco states.

#### 2.2.2 Pre-trained backbone

To build a deep learning benchmark and verify the efficacy of deep learning networks for the tobacco leaves states recognition, four extensively employed deep neural networks pre-trained on ImageNet were utilized as backbone network, including VGG19, ResNet-152, ViT, Swin-Transformer and Swin-Transformer v2 (Simonyan and Zisserman, 2014; He et al., 2016; Dosovitskiy et al., 2020; Liu et al., 2021; 2022). VGG19 is a profound convolutional neural network comprising 16 convolution layers and 3 fully connected layers. ResNet-152 is an exceptionally deep convolutional neural network, reaching a depth of up to 152 layers, made possible by employing skip connections to bypass certain layers. ViT-Large is a model that applies the transformer architecture, which has demonstrated impressive performance in the field of computer vision recently. The Swin Transformer is a hierarchical vision model engineered for efficient image recognition. It utilizes non-overlapping windows and self-attention within each window to process images at multiple scales. Swin-Transformer V2 enhances this approach with innovations like scaled cosine attention, post- normalization, and a log-spaced continuous position bias, boosting stability, scalability, and overall performance.

These pre-trained backbones extract highly discriminative image features by leveraging the information learned before and further fine-tuning the parameters on the proposed datasets. An evaluation of the predictive accuracy of these four networks in determining the state of tobacco was conducted. To further enhance their performance, Swin-Transformer v2 was incorporated as the backbone network and its core components including the following two aspects.

##### 2.2.2.1 The attention mechanism

In the Swin-Transformer v2, the attention mechanism is a crucial component (Vaswani et al., 2017). It performs multiple attention operations to extract highly discriminative features. Given N image patches within an image and their corresponding features F ∈ RN×d′ (the process to obtain F will be detailed in the subsequent paragraph), the operation of the self-attention mechanism is as follows:

First, the query (Q), key (K), and value (V) are computed using a linear transformation with the trainable weight W ∈ R^d′×3d^ (Equations 1):


(1) Q, K, V =WF

where Q, K, V ∈ R^N×d^, W represents the weight matrix of a linear layer, where *d^′^* is the input channel dimension of the feature F ∈ K V × *d^′^*, *d* is the output channel dimension of the linear layer. Then, the attention mechanism is applied to extract the output feature (Equations 2, 3):


(2) Attention(Q, K, V)=SoftMax(cos(Q, K)/γ+B)V

(3) $$SoftMax{\left(X\right)}_{i}=\frac{exp\left(X_{i}\right)}{\sum_{j=1}^{N}exp\left(X_{j}\right)}$$

where cos(Q, K) ∈ R^N×N^ is the pair-wise cosine similarity, γ is a learnable scalar and *N* denotes the number of image patches. The B ∈ R^N×N^ serves as a relative positional encoding which is predicted by two trainable fully connected layers g (Equation 4):


(4) B(ΔX, ΔY)=g(ΔX, ΔY)

##### 2.2.2.2 Patch splitting and merging

The attention mechanism is implemented within the image patches, which are derived from segmenting an input image into non-overlapping patches. Each patch is regarded as an individual unit, referred to as a “token”. Then a linear projection transforms each token to the token features F. The token features are subsequently fed into numerous layers of the Swin-Transformer V2. Each layer is designed to extract and refine the information embedded within the tokens. This refinement process involves a series of operations that integrate attention mechanisms. As the tokens progress further into the depths of the network, a method known as patch merging is employed. This method reduces the number of tokens by integrating the features of neighboring patches. The result is an ensemble of tokens, thereby effectively establishing a hierarchical representation of the initial image.

#### 2.2.3 Fourier filter module

**Figure 4:**
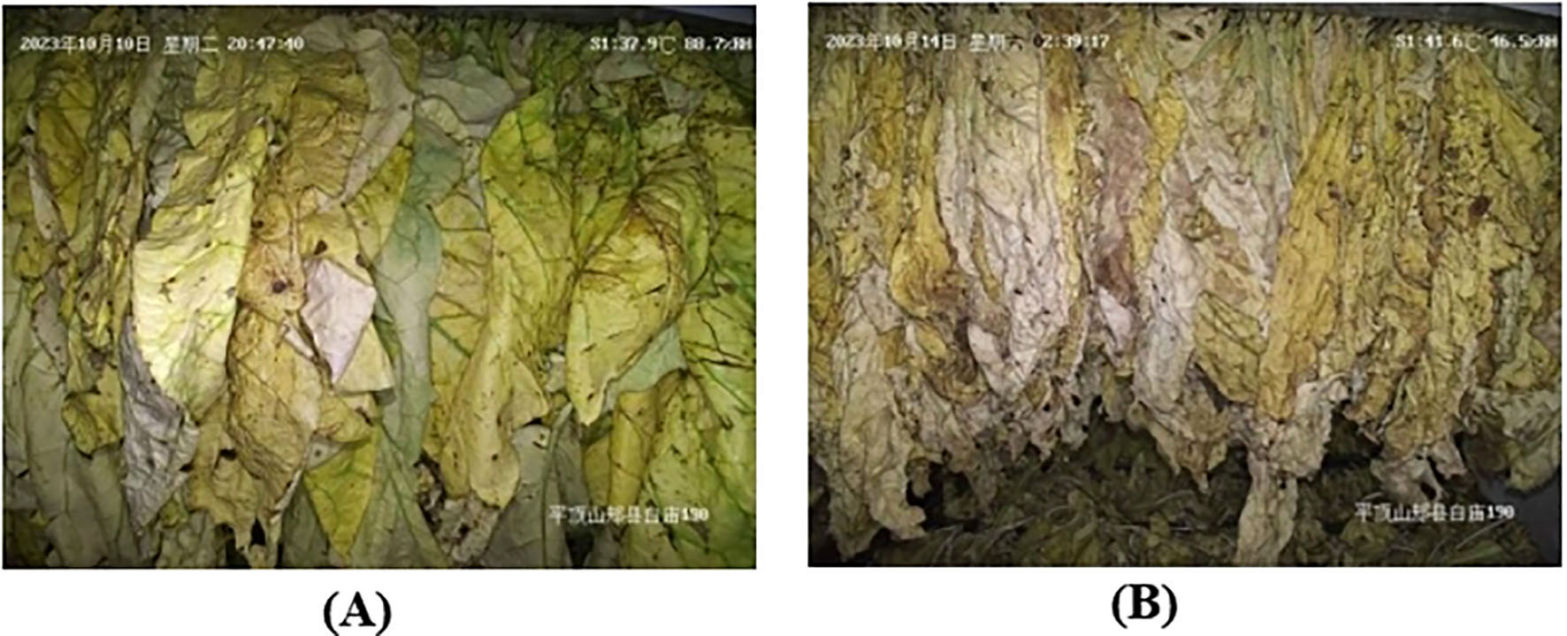
Images of tobacco leaves with **(A)** low drying and **(B)** high drying degrees.

The prediction of drying is more dependent on the morphological characteristics of the tobacco leaves compared to the prediction of yellowing and browning. As depicted in **Figure 4**, images of tobacco leaves with a higher degree of drying display a greater number of wrinkles, which are associated with the high-frequency information in the image’s Fourier spectrum. As the drying process progresses, the formation of surface wrinkles on tobacco leaves increases, thereby amplifying the high-frequency intensity in the corresponding images.

**Figure 5:**
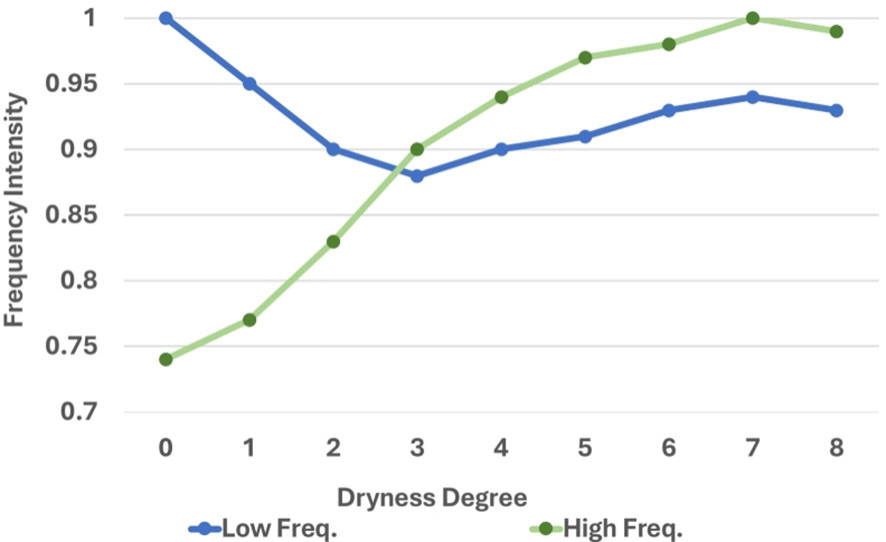
Statistical (normalized) high/low frequency intensity of images with different drying degrees.

To validate this hypothesis, the average high-frequency and low-frequency intensities of images with different degrees of drying within the training set were computed. The results, as illustrated in **Figure 5**, revealed that the average high-frequency intensity of the corresponding image exhibits an upward trend as the degree of drying increases. This suggested a correlation between the image’s frequency domain information and its degree of drying. Consequently, the Fourier spectrum was incorporated as information into the network and a Fourier filter was construct to aid in the prediction of the degrees of drying.

Specifically, the image was converted into a gray-scale image at first and then its two-dimensional Fourier spectrum was calculated (Equation 5):


(5) $$F\left(u,v\right)=\sum_{m=0}^{H-1}\sum_{n=0}^{W-1}f\left(m,n\right)e^{-j2\pi \left(um/H+vn/W\right)}$$

where F is the Fourier spectrum with the same shape as the input image. The H and W are the height and width, respectively. Then the real and imaginary components of each frequency position within this matrix are utilized as input for the neural network. Where the values of real and imaginary components are represented as separate image channels. Four-layer convolutional layers are constructed to extract spectral features Fspect from this input. This approach enables the effective capture of intricate patterns within the Fourier spectrum, thereby enhancing the robustness of the drying prediction.

#### 2.2.4 Common color filter module

In conjunction with the Fourier Filter module, which is primarily designed to augment the prediction of drying, an additional module denoted as the Common Color Filter was introduced. This module was specifically engineered to enhance the prediction accuracy of the states intrinsically tied to the color of the tobacco leaves. However, the image signal frequently encompasses elements beyond the mere color of the tobacco, the presence of noise color could potentially compromise the final prediction. Therefore, it is important to eliminate as many noisy pixels as possible to mitigate color interference. To address this challenge, the characteristic that the tobacco in the bulk curing barn is densely arranged and typically occupies a consistent position were exploited. This strategy aided in the effective reduction of noise and enhanced the accuracy of the proposed model.

**Figure 6:**
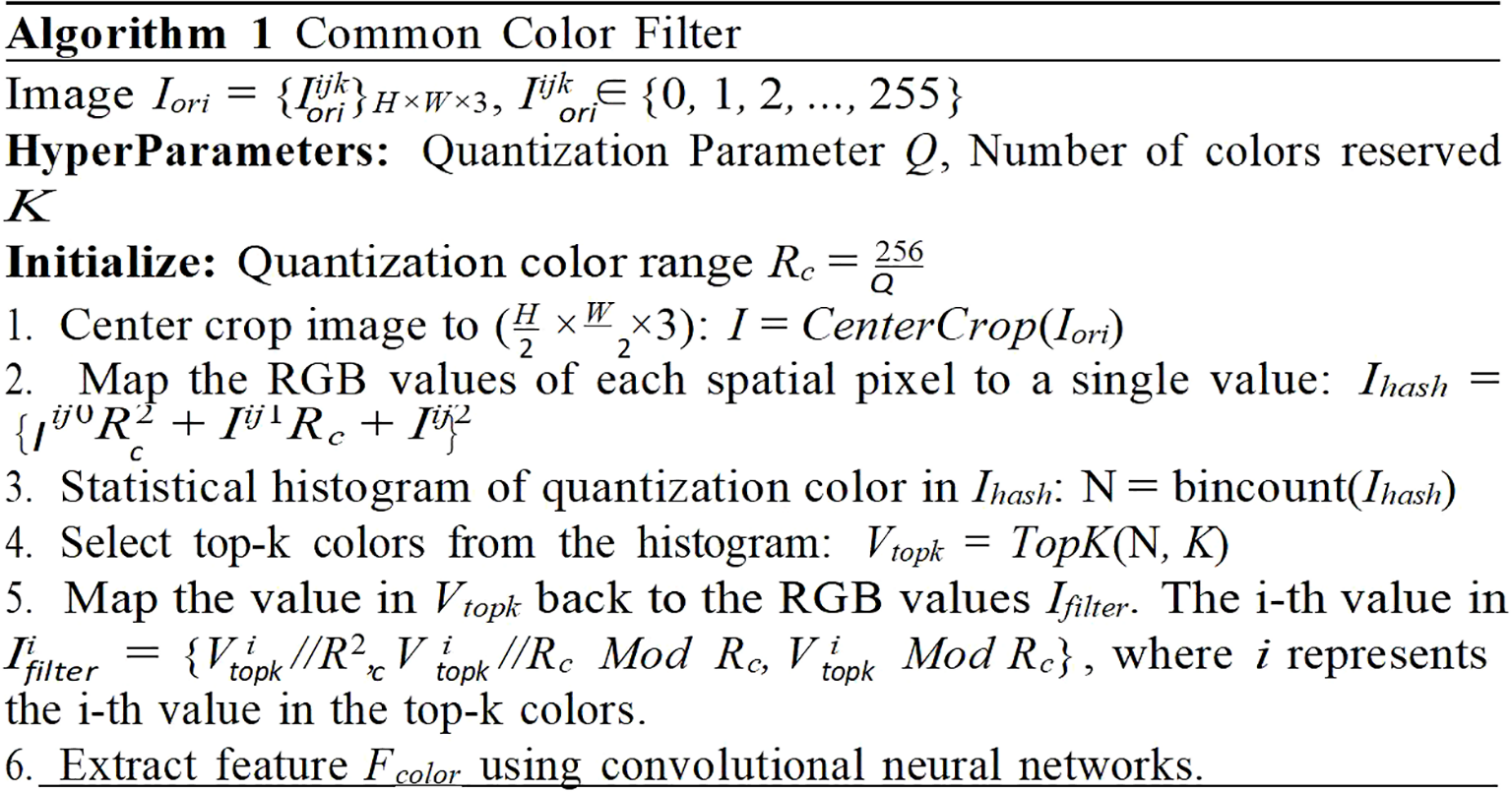
Implementation procedure of filtering algorithm.

**Figure 7:**
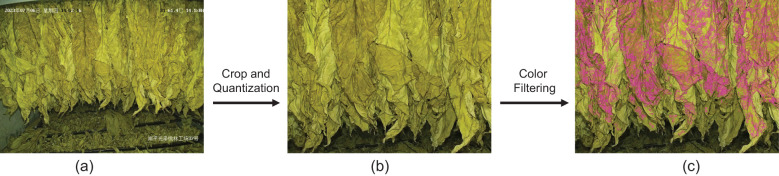
Visualization of filtering process: **(A)** input image, **(B)** quantized and center- cropped image and **(C)** after histogram filtering. The pixel positions corresponding to the retained colors (shown in purple) primarily focus on the tobacco leaves rather than other background areas.

As depicted in **Figure 6**, this algorithm filtered out the most common colors in an image and extracts relevant features for subsequent use. It accomplished this through a series of steps. Firstly, it applied a center-cropping technique to the image. This process focused on the central part of the image, which contained the most important information and reduced the impact of potential noise from the image’s periphery. Next, it quantized the color space. Quantization was a process that reduced the number of distinct colors used in an image, while still maintaining its overall visual construction. This step can reduce the size of the color space and the computation load when calculating the color histogram. Following this, the most common colors were selected. These colors are shown in **Figure 7**, it depicted typically the tobacco leaves. By focusing on these colors, the algorithm can more accurately predict the state of yellowing and browning. Finally, the common color feature Fcolor was extracted using a single fully-connected layer.

### 2.3 Preprocessing and evaluation metrics

During training, input images were resized to 384×384, the supported input size of our image backbone. Data augmentation was applied using the RandAugment method with a magnitude of 9 and a standard deviation of 0.5.

The primary evaluation metric employed to assess the algorithms’ performance was top-1 accuracy, expressed as a percentage. Specifically, the network’s prediction probability for the most likely state was selected as the predicted result and compared with the ground-truth label. The accuracy is then calculated using the (Equation 6):


(6) $$Accuracy=\frac{\#TruePostive}{\#TruePositive+\#FalsePostive}$$

where *#TruePostive* represents instances where the model accurately predicts the positive class, *#FalsePostive* denotes instances of incorrect predictions by the model, the symbol “#” indicates the number of corresponding instances or categories. The individual prediction accuracy for three different tobacco leaves states as well as their average accuracy were separately evaluated.

## 3 Results and discussion

### 3.1 Accuracy of tobacco leaves state prediction

#### 3.1.1 Comparison with traditional algorithms

The performance of three traditional algorithms on the same task, including K nearest neighbor (KNN), support vector machines (SVM), and random forest (RF), was compared with our deep-learning method (Cover and Hart, 1967; Hearst et al., 1998; Breiman, 2001). Three distinct predicted states of tobacco leaves and hyperparameters for each method are shown in **Tables 2**, **3**, respectively.

**Table 2:** The prediction accuracy of different traditional methods.

Methods		Accuracy			
		Yellowing	Browning	Drying	All
Traditional	KNN	66.5	84.5	54.6	68.5
	RF	71.2	84.3	64.6	73.4
	SVM	73.9	85.7	64.4	74.7
Deep learning	Ours	**83.0**	**90.5**	**75.6**	**83.0**

Bold font is the best result in each experiment.

**Table 3:** The hyperparameters of different traditional methods.

Methods	Hyperparameters	Value
KNN	neighbors	5
	distance type	Frobenius norm
SVM	C	1
	kernel	Linear
	Penalty term	L2
	loss	squared hinge
RF	Number of trees	100
	Split criterion	Gini impurity
	bootstrap	True
	n_bins	1024

Firstly, the deep-learning method significantly outperformed the three traditional methods, highlighting the potential benefits of using deep learning for this task. Secondly, the KNN method performed well in predicting browning degree by directly computing the difference between two images as the norm, and was ineffective for yellowing degree and drying degree. This suggested that the prediction of browning degree relied more on the color information within the image.

#### 3.1.2 Comparison with deep learning algorithms

As shown in **Tables 4**, **5**, Swin-Transformer v2-Large achieved state-of-the-art performance among all other single backbones and benefited from a larger in-put size. The method proposed in this study further enhanced Swin-Transformer v2-Large’s performance, demonstrating a higher accuracy with an average accuracy of 83.0%. To verify the effectiveness of the two proposed modules (FFM and CCFM), The new method was also implemented based on the Swin-Transformer-Large as the pre-trained backbone network. It can be observed that after integrating FFM and CCFM modules with the Swin-Transformer, an improvement in accuracy was achieved (81.8% vs. 81.6%). This indicated that these modules possessed a certain degree of robustness.

**Table 4:** The prediction accuracy of different deep learning methods.

Methods	Resolution	Pre-trained	Accuracy			
			Yellowing	Browning	Drying	All
VGG-19	224×224	ImageNet1K	79.2	87.7	70.6	79.1
ResNet-152	224×224	ImageNet1K	78.7	88.7	70.1	79.2
ViT-Large	224×224	ImageNet22K	80.7	90.1	71.4	80.7
Swin-Base	224×224	ImageNet1K	80.8	89.3	73.0	81.0
ConvNeXt V2	224×224	ImageNet22K	81.5	90.1	74.0	81.9
Swin-Base	224×224	ImageNet22K	81.1	89.7	73.3	81.4
Swin-Large	224×224	ImageNet22K	81.3	90.2	73.2	81.6
Ours(Swin-Large)	224×224	ImageNet22K	81.3	90.3	73.9	81.8
SwinV2-Large	256×256	ImageNet22K	82.2	90.2	73.9	82.1
SwinV2-Large	384×384	ImageNet22K	82.6	89.6	75.0	82.4
Ours(SwinV2-Large)	384×384	ImageNet22K	**83.0**	**90.5**	**75.6**	**83.0**

Bold font is the best result in each experiment.

**Table 5:** The hyperparameters of our methods.

Hyperparameters	Value
warmup learning rate	2e-8
base learning rate	2e-4
end learning rate	2e-7
batch size	8
decay-epoch	5
learning rate schedule	cosine
optimizer	AdamW
drop-path	0.1
gradient clip	1

To further verify the impact of image resolution and pre-trained image datasets on the accuracy of tobacco condition recognition, experiments with different parameters based on Swin-Transformer and Swin-Transformer v2 were conducted. The results indicated that the accuracy of tobacco leaves states recognition can benefit from being pre-trained on a larger image dataset (i.e., ImageNet-22k), even if it was not directly related to tobacco leaves. Higher input resolution can also improve prediction accuracy, suggesting that it is possible to further enhance the accuracy of tobacco leaf recognition by increasing the input resolution. Moreover, this method required low computational cost and provided fast output, with a less than 2GB of GPU memory for inference during testing and an average prediction time of under 0.5 seconds per image.

### 3.2 Ablation experiment of the proposed component

Ablation experiments were conducted to demonstrate the effectiveness of the three proposed components in this paper. As depicted in **Table 6**, when compared to the standalone backbone model, the Fourier Filter Module (FFM) contributed an absolute improvement of 1.0% in drying prediction accuracy, underscoring its efficacy in enhancing drying prediction. Similarly, the Common Color Filter Module (CCFM) accounted for an absolute increase of 0.8% in browning prediction, affirming its utility in tasks significantly influenced by color attributes. Moreover, as indicated in the final row, the synergistic integration of both modules lead to further enhancements in prediction precision, thereby confirming that their combined application can substantially bolster overall performance.

**Table 6:** The ablation of the proposed component.

Backbone	FFM	CCFM	Accuracy			
			Yellowing	Browning	Drying	All
✓			82.6	89.6	75.0	82.4
✓	✓		83.0	89.5	76.0	82.8
✓		✓	82.5	90.4	75.4	82.8
✓	✓	✓	83.0	90.5	75.6	83.0

### 3.3 Effectiveness of the hyper-parameters in the common color filter module

The effectiveness of the two hyper-parameters, quantization parameter Q and the number of colors reserved K, related to the common color filter module were evaluated. Different values of Q and K were chosen. The experimental results are shown in **Table 7**. The optimum value was obtained when Q = 4 and K = 20. When Q = 2, the color granularity became smaller and showed higher performance in predicting browning and yellowing, and the drying slightly decreased. One possible reason was that this fine color information dominated the feature extraction. Under the same Q, sampling with different K will also lead to different results, indicating that the balance the situations of insufficient sampling and excessive noise sampling through K was required.

**Table 7:** The ablation of the hyper-parameters in the common color filter module.

Q	K	Accuracy			
		Yellowing	Browning	Drying	All
4	10	82.7	89.8	75.3	82.6
4	20	83.0	**90.5**	**75.6**	**83.0**
4	30	82.7	90.1	75.3	82.7
8	20	83.1	90.2	74.9	82.8
2	20	**83.2**	**90.5**	75.0	82.9

Bold font is the best result in each experiment.

### 3.4 Effectiveness of production area independent prediction

The large scale dataset comprised images acquired across various production areas, sensor discrepancies, tobacco varieties and ecological environmental variations can all contribute to inherent difference of images. While a unified training approach was straightforward and widely adopted, the impact of area specificity on the recognition of tobacco states was also explored in this study. Three representative production areas with substantial data volume were selected and individual training processes were conducted. In addition, the integrated training model in all areas was used to make separate predictions for these areas and compared them with the results of the separately trained model. This allowed assessments on area-specific models’ effectiveness and their practicality for different tobacco production areas.

The experiment was conducted in three distinct production areas, labeled as A, B, and C. Each bulk curing barn employed an image device, with the corresponding data statistics presented in **Table 8**. As shown in **Table 9**, it was evident across three distinct areas that the integrated model outperformed models trained individually for each area in terms of prediction accuracy. This suggested that despite the inherent difference present in images from different areas, the model can still leverage a larger image dataset to enhance its performance, surpassing that of models trained individually in each area.

**Table 8:** Data statistics of three typical production areas.

Production area	A	B	C
Training data	1.2k	2.9k	1.2k
Test data	0.5k	1.2k	0.5k
All data	1.7k	4.1k	1.7k

**Table 9:** The comparison of training of different areas separately.

Area	Individual model				Integrated model			
	Yellowing	Browning	Drying	All	Yellowing	Browning	Drying	All
All	–	–	–	–	83.0	90.5	75.6	83.0
A	84.7	87.1	76.9	82.9	84.8	87.6	78.7	83.7
B	82.2	90.9	77.7	83.6	83.7	90.6	77.5	84.0
C	86.0	89.0	77.8	84.3	84.6	90.5	79.5	84.9

### 3.5 Visualization of the confusion matrix

**Figure 8:**
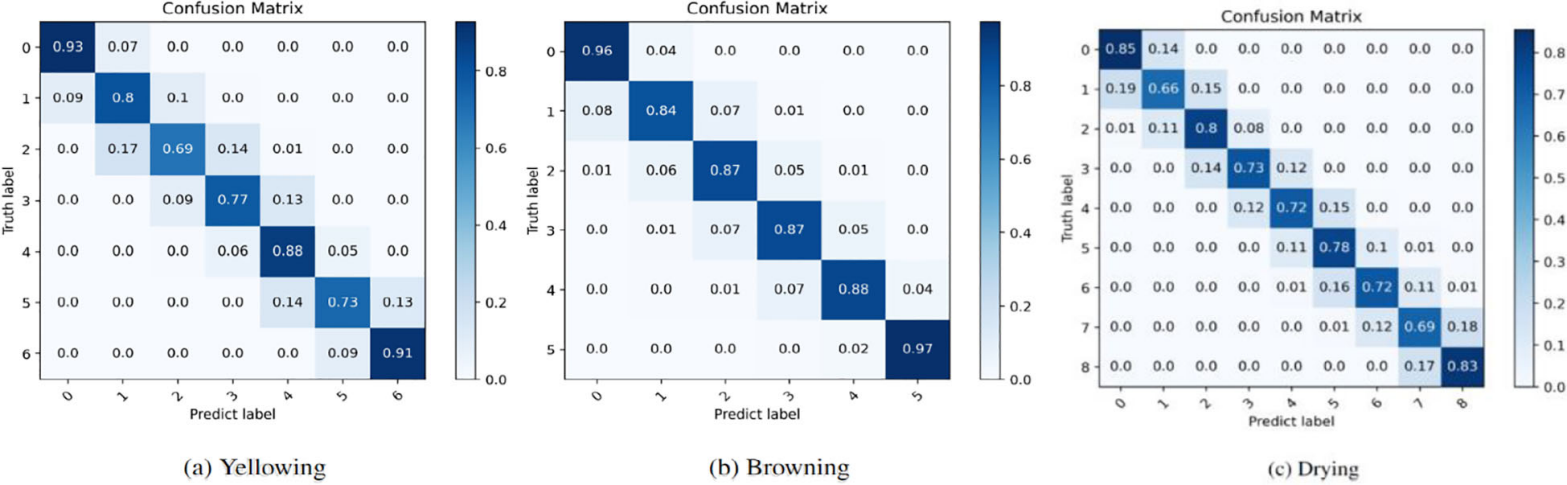
The confusion matrices for the prediction of the **(A)** Yellowing, **(B)** Browning and **(C)** Drying states of the tobacco leaves.

As shown in **Figure 8**, a deeper analysis of the confusion matrix corresponding to the three predicted states was conducted. The results illustrated that the proposed method demonstrated remarkable predictive accuracy. For each ground-truth label across the three states, the majority of the predicted labels align with either the ground-truth labels or their neighboring labels (the cumulative probability for these exceeds 99%), which was reasonable given the inherent difficulty in distinguishing between neighboring state image features due to their significant similarity. It is difficult to capture the morphological changes at the critical stage of the tobacco leaf curing process. Even if experienced experts make judgments, they may still misjudge. The prediction of the browning degree (**Figure 8b**) demonstrates high accuracy across all degrees. However, further improvements are still required in the prediction of the middle degrees of yellowing (**Figure 8a**) and drying (**Figure 8c**). Generally, the accuracy of the proposed method complete recognition is 83%, the accuracy rate of adjacent stages is more than 99%, and the fault tolerance rate is within ±1 stage, which has little impact on the curing quality during the actual curing process.

### 3.6 Visualization of the gradient.

**Figure 9:**
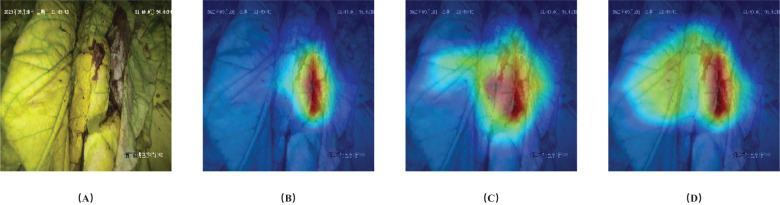
Visualization of gradients with respect to ground truth labels. **(A)** Original image, **(B)** Browning, **(C)** Drying and **(D)** Yellowing states.

In **Figure 9**, GradCam (Selvaraju et al., 2017) was used to visualize the gradients for each state. The results showed that in this example, the model proposed in this study focused primarily on the brown parts of the image when predicting browning degree, on the petiole when predicting drying degree, and on larger areas of leaf content when predicting yellowing degree. This is similar to the reference positions and standards that people use to judge the three states of tobacco leaves during the curing process.

## 4 Conclusions

In this study, an large-scale dataset including 17,420 images of tobacco leaves from 10 main production areas in China was developed, with a specific emphasis on recognizing the degrees of yellowing, browning, and drying. This is a large-scale dataset specifically dedicated to the morphological recognition of the states of tobacco leaves within an actual bulk curing barn setting. A deep learning benchmark was then established for this dataset using various deep learning networks. To further enhance the predictive performance of the deep backbone network, an efficient deep learning method was proposed, including Fourier filter module and common color filter module. This method integrated the spectral characteristics of tobacco leaves images and filters out color noise, which effectively enhanced the accuracy of our model with prediction accuracy for the yellowing degree, browning degree, and drying degree were 83.0%, 90.5%, and 75.6% respectively. The high overall average accuracy with a value of 83.0% and the availability and feasibility in different production areas have demonstrated the superior performance of the proposed method in this study, which provides a solid foundation for future research in this area.

## Data Availability

The raw data supporting the conclusions of this article will be made available by the authors, without undue reservation.
